# The non-negative matrix factorization toolbox for biological data mining

**DOI:** 10.1186/1751-0473-8-10

**Published:** 2013-04-16

**Authors:** Yifeng Li, Alioune Ngom

**Affiliations:** 1 School of Computer Science, University of Windsor, Windsor, Ontario, Canada

**Keywords:** Non-negative matrix factorization, Clustering, Bi-clustering, Feature extraction, Feature selection, Classification, Missing values

## Abstract

**Background:**

Non-negative matrix factorization (NMF) has been introduced as an important method for mining biological data. Though there currently exists packages implemented in R and other programming languages, they either provide only a few optimization algorithms or focus on a specific application field. There does not exist a complete NMF package for the bioinformatics community, and in order to perform various data mining tasks on biological data.

**Results:**

We provide a convenient MATLAB toolbox containing both the implementations of various NMF techniques and a variety of NMF-based data mining approaches for analyzing biological data. Data mining approaches implemented within the toolbox include data clustering and bi-clustering, feature extraction and selection, sample classification, missing values imputation, data visualization, and statistical comparison.

**Conclusions:**

A series of analysis such as molecular pattern discovery, biological process identification, dimension reduction, disease prediction, visualization, and statistical comparison can be performed using this toolbox.

## Background

*Non-negative matrix factorization* (NMF) is a matrix decomposition approach which decomposes a non-negative matrix into two low-rank non-negative matrices [1]. It has been successfully applied in the mining of biological data.

For example, Ref. [2,3] used NMF as a clustering method in order to discover the metagenes (i.e., groups of similarly behaving genes) and interesting molecular patterns. Ref. [4] applied *non-smooth NMF* (NS-NMF) for the biclustering of gene expression data. *Least-squares NMF* (LS-NMF) was proposed to take into account the uncertainty of the information present in gene expression data [5]. Ref. [6] proposed kernel NMF for reducing dimensions of gene expression data.

Many authors indeed provide their respective NMF implementations along with their publications so that the interested community can use them to perform the same data mining tasks respectively discussed in those publications. However, there exists at least three issues that prevent NMF methods from being used by the much larger community of researchers and practitioners in the data mining, biological, health, medical, and bioinformatics areas. First, these NMF softwares are implemented in diverse programming languages, such as R, MATLAB, C++, and Java, and usually only one optimization algorithm is provided in their implementations. It is inconvenient for many researchers who want to choose a suitable NMF method or mining task for their data, among the many different implementations, which are realized in different languages with different mining tasks, control parameters, or criteria. Second, some papers only provide NMF optimization algorithms at a basic level rather than a data mining implementation at a higher level. For instance, it becomes hard for a biologist to fully investigate and understand his/her data when performing clustering or bi-clustering of his data and then visualize the results; because it should not be necessary for him/her to implement these three data mining methods based on a basic NMF. Third, the existing NMF implementations are application-specific, and thus, there exists no systematic NMF package for performing data mining tasks on biological data.

There currently exists NMF toolboxes (which we discuss in this paragraph), however, none of them addresses the above three issues altogether.

*NMFLAB*[7] is MATLAB toolbox for signal and image processing which provides a user-friendly interface to load and process input data, and then save the results. It includes a variety of optimization algorithms such as multiplicative rules, exponentiated gradient, projected gradient, conjugate gradient, and quasi-Newton methods. It also provides methods for visualizing the data signals and their components, but does not provide any data mining functionality. Other NMF approaches such as semi-NMF and kernel NMF are not implemented within this package.

*NMF:DTU Toolbox*[8] is a MATLAB toolbox with no data mining functionalities. It includes only five NMF optimization algorithms, such as multiplicative rules, projected gradient, probabilistic NMF, alternating least squares, and alternating least squares with *optimal brain surgery* (OBS) method.

*NMFN: Non-negative Matrix Factorization*[9] is an R package similar to *NMF:DTU* but with few more algorithms.

*NMF: Algorithms and framework for Nonnegative Matrix Factorization*[10] is another R package which implements several algorithms and allows parallel computations but no data mining functionalities.

*Text to Matrix Generator (TMG)* is a MATLAB toolbox for text mining only.

Ref. [11] provides a NMF plug-in for BRB-ArrayTools. This plug-in only implements the standard NMF and semi-NMF and for clustering gene expression profiles only.

*Coordinated Gene Activity in Pattern Sets* (CoGAPS) [12] is a new package implemented in C++ with R interface. In this package, the *Bayesian decomposition* (BD) algorithm is implemented and used in place of the NMF method for factorizing a matrix. Statistical methods are also provided for the inference of biological processes. CoGAPS can give more precise results than NMF methods [13]. However, CoGAPS uses a Markov chain Monte Carlo (MCMC) scheme for estimating the BD model parameters, which is slower than the NMFs optimization algorithms implemented with the block-coordinate gradient descent scheme.

In order to address the lack of data mining functionalities and generality of current NMF toolboxes, we propose a general NMF toolbox in MATLAB which is implemented in two levels. The basic level is composed of the different variants of NMF, and the top level consists of the diverse data mining methods for biological data. The contributions of our toolbox are enumerated in the following: 

1. The NMF algorithms are relatively complete and implemented in MATLAB. Since it is impossible and unnecessary to implement all NMF algorithms, we focus only on well-known NMF representatives. This repository of NMFs allows users to select the most suitable one in specific scenarios.

2. Our NMF toolbox includes many functionalities for mining biological data, such as clustering, bi-clustering, feature extraction, feature selection, and classification.

3. The toolbox also provides additional functions for biological data visualization, such as heat-maps and other visualization tools. They are pretty helpful for interpreting some results. Statistical methods are also included for comparing the performances of multiple methods.

The rest of this paper is organized as below. The implementations of the basis level are first discussed in the next section. After that, examples of implemented data mining tasks at a high level are described. Finally, we conclude this paper and give possible avenues for future research directions.

## Implementation

As mentioned above, this toolbox is implemented at two levels. The fundamental level is composed of several NMF variants and the advanced level includes many data mining approaches based on the fundamental level. The critical issues in implementing these NMF variants are addressed in this section. Table 1 summarizes all the NMF algorithms implemented in our toolbox. Users (researchers, students, and practitioners) should use the command help nmfrule, for example, in the command line, for help on how to select a given funtion and set its parameters.

**Table 1:** Algorithms of NMF variants

**Function**	**Description**
nmfrule	The standard NMF optimized by gradient-descent-based multiplicative rules.
nmfnnls	The standard NMF optimized by NNLS active-set algorithm.
seminmfrule	Semi-NMF optimized by multiplicative rules.
seminmfnnls	Semi-NMF optimized by NNLS.
sparsenmfnnls	Sparse-NMF optimized by NNLS.
sparsenmfNNQP	Sparse-NMF optimized by NNQP.
sparseseminmfnnls	Sparse semi-NMF optimized by NNLS.
kernelnmfdecom	Kernel NMF through decomposing the kernel matrix of input data.
kernelseminmfrule	Kernel semi-NMF optimized by multiplicative rule.
kernelseminmfnnls	Kernel semi-NMF optimized by NNLS.
kernelsparseseminmfnnls	Kernel sparse semi-NMF optimized by NNLS.
kernelSparseNMFNNQP	Kernel sparse semi-NMF optimized by NNQP.
convexnmfrule	Convex-NMF optimized by multiplicative rules.
kernelconvexnmf	Kernel convex-NMF optimized by multiplicative rules.
orthnmfrule	Orth-NMF optimized by multiplicative rules.
wnmfrule	Weighted-NMF optimized by multiplicative rules.
sparsenmf2rule	Sparse-NMF on both factors optimized by multiplicative rules.
sparsenmf2nnqp	Sparse-NMF on both factors optimized by NNQP.
vsmf	Versatile sparse matrix factorization optimized by NNQP and *l*_1_QP.
nmf	The omnibus of the above algorithms.
computeKernelMatrix	Compute the kernel matrix k(A,B) given a kernel function.

### Standard-NMF

The *standard-NMF* decomposes a non-negative matrix $X\in {\mathbb{R}}^{m\times n}$ into two non-negative factors $A\in {\mathbb{R}}^{m\times k}$ and $Y\in {\mathbb{R}}^{k\times n}$ (where *k*< min{*m*,*n*}), that is

(1) $$X_{+}=A_{+}Y_{+}+E,$$

where, ***E*** is the error (or residual) and ***M***_+_ indicates the matrix ***M*** is non-negative. Its optimization in the Euclidean space is formulated as

(2) $$\underset{A,Y}{\min}\frac{1}{2}∥X-AY{∥}_{F}^{2},\text{subject to},A,Y\geq 0.$$

Statistically speaking, this formulation is obtained from the log-likelihood function under the assumption of a Gaussian error. If multivariate data points are arranged in the columns of ***X***, then ***A*** is called the *basis matrix* and ***Y*** is called the *coefficient matrix*; each column of ***A*** is thus a *basis vector*. The interpretation is that each data point is a (sparse) non-negative linear combination of the basis vectors. It is well-known that the optimization objective is a non-convex optimization problem, and thus, *block-coordinate descent* is the main prescribed optimization technique for such problem. Multiplicative update rules were introduced in [14] for solving Equation (2). Though simple to implement, this algorithm is not guaranteed to converge to a stationary point [15]. Essentially the optimizations above, with respect to ***A*** and ***Y***, are *non-negative least squares* (NNLS). Therefore we implemented the alternating NNLS algorithm proposed in [15]. It can be proven that this algorithm converges to a stationary point. In our toolbox, functions nmfrule and nmfnnls are the implementations of the two algorithms above.

### Semi-NMF

The standard NMF only works for non-negative data, which limits its applications. Ref. [16] extended it to s*emi-NMF* which removes the non-negative constraints on the data ***X*** and basis matrix ***A***. It can be expressed in the following equation:

(3) $$\underset{A,Y}{\min}\frac{1}{2}∥X-AY{∥}_{F}^{2},\text{subject to}\;Y\geq 0.$$

Semi-NMF can be applied to the matrix of mixed signs, therefore it expands NMF to many fields. However, the gradient-descent-based update rule proposed in [16] is slow to converge (implemented in function seminmfrule in our toolbox). Keeping ***Y*** fixed, updating ***A*** is a least squares problem which has an analytical solution

(4) $$\begin{array}{ll} A=XY^{T}{\left(YY^{T}\right)}^{-1}=XY^{‡}, & \; \end{array}$$

where ***Y***^*‡*^=***Y***^T^(***YY***^T^)^−1^ is Moore-Penrose pseudoinverse. Updating ***Y*** while fixing ***A*** is a NNLS problem essentially as above. Therefore we implemented the fast NNLS based algorithm to optimize semi-NMF in function seminmfnnls.

### Sparse-NMF

The standard NMF and semi-NMF have the issues of scale-variance and non-unique solutions, which imply that the non-negativity constrained on the least squares is insufficient in some cases. Sparsity is a popular regularization principle in statistical modeling [17], and has already been used in order to reduce the non-uniqueness of solutions and also and enhance interpretability of the NMF results. The *sparse-NMF* proposed in [3] is expressed in the following equation

(5) $$\begin{array}{ll} \underset{A,Y}{\min}\frac{1}{2}∥X-AY{∥}_{F}^{2}+\frac{\eta }{2}∥A{∥}_{F}^{2}+\frac{\lambda }{2}\overset{n}{\underset{i=1}{\sum }}∥y_{i}{∥}_{1}^{2}\;\\ \text{subject to}\;A,Y\geq 0,\; & \; \end{array}$$

where, ***y***_*i*_ is the *i*-th column of ***Y***. From the Bayesian perspective, this formulation is obtained from the log-posterior probability under the assumptions of Gaussian error, Gaussian-distributed basis vectors, and Laplace-distributed coefficient vectors. Keeping one matrix fixed and updating the other matrix can be formulated as a NNLS problem. In order to improve the interpretability of the basis vectors and speed up the algorithm, we implemented the following model instead:

(6) $$\begin{array}{ll} \underset{A,Y}{\min}\frac{1}{2}∥X-AY{∥}_{F}^{2}+\lambda \overset{n}{\underset{i=1}{\sum }}∥y_{i}{∥}_{1}\;\\ \text{subject to}\;A,Y\geq 0,\;\\ \;∥a_{i}{∥}_{2}^{2}=1,\;i=1,\cdots \;,\mathrm{k.}\; & \; \end{array}$$

We optimize this using three alternating steps in each iteration. First, we optimize the following task:

(7) $$\begin{array}{ll} \underset{Y}{\min}\frac{1}{2}∥X-AY{∥}_{F}^{2}+\lambda \overset{n}{\underset{i=1}{\sum }}∥y_{i}{∥}_{1}\;\\ \text{subject to}\;Y\geq 0.\; & \; \end{array}$$

then, ***A*** is updated as follows:

(8) $$\begin{array}{ll} \underset{A}{\min}\frac{1}{2}∥X-AY{∥}_{F}^{2}\;\\ \text{subject to}\;A\geq 0.\; & \; \end{array}$$

and then, the columns of ***A*** are normalized to have unit *l*_2_ norm. The first and second steps can be solved using *non-negative quadratic programming* (NNQP), whose general formulation is

(9) $$\begin{array}{ll} \underset{Z}{\min}\overset{n}{\underset{i=1}{\sum }}\frac{1}{2}z_{i}^{T}Hz_{i}+g_{i}^{T}z_{i}+c_{i}\;\\ \text{subject to}\;Z\geq 0,\; & \; \end{array}$$

where, ***z***_*i*_ is the *i*-th column of the variable matrix ***Z***. It is easy to prove that NNLS is a special case of NNQP. For example, Equation (7) can be rewritten as

(10) $$\begin{array}{ll} \underset{Y}{\min}\overset{n}{\underset{i=1}{\sum }}\frac{1}{2}y_{i}^{T}(A^{T}A)y_{i}+{\left(\lambda -A^{T}x_{i}\right)}^{T}y_{i}+x_{i}^{T}x_{i}\;\\ \text{subject to}\;Y\geq 0.\; & \; \end{array}$$

The implementations of the method in [3] and our method are given in functions sparsenmfnnls and sparseNMFNNQP, respectively. We also implemented the sparse semi-NMF in functionl sparseseminmfnnls.

### Versatile sparse matrix factorization

When the training data ***X*** is of mixed signs, the basis matrix ***A*** is not necessarily constrained to be non-negative; this depends on the application or the intentions of the users. However, without non-negativity, ***A*** is not sparse any more. In order to obtain sparse basis matrix ***A*** for some analysis, we may use *l*_1_-norm on ***A*** to induce sparsity. The drawback of *l*_1_-norm is that correlated variables may not be simultaneously non-zero in the *l*_1_-induced sparse result. This is because *l*_1_-norm is able to produce sparse but non-smooth results. It is known that *l*_2_-norm is able to obtain smooth but non-sparse results. When both norms are used together, then correlated variables can be selected or removed simultaneously [18]. When smoothness is required on ***Y***, we may also use *l*_2_-norm on it in some scenarios. We thus generalize the aforementioned NMF models into a versatile form as expressed below

(11) $$\begin{array}{ll} \underset{A,Y}{\min}f(A,Y)=\frac{1}{2}∥X-AY{∥}_{F}^{2}+\overset{k}{\underset{i=1}{\sum }}(\frac{\alpha_{2}}{2}∥a_{i}{∥}_{2}^{2}\; & \;\\ \;+\alpha_{1}∥a_{i}{∥}_{1})+\overset{n}{\underset{i=1}{\sum }}(\frac{\lambda_{2}}{2}∥y_{i}{∥}_{2}^{2}+\lambda_{1}∥y_{i}{∥}_{1})\;\\ \text{subject to}\;\left\{\begin{array}{ll} A\geq 0\; & \text{i.e., if}\;\;t_{1}=1\\ Y\geq 0\; & \text{i.e., if}\;\;t_{2}=1\\ \; \end{array}\right),\; & \; \end{array}$$

where, parameters: *α*_1_≥0 controls the sparsity of the basis vectors; *α*_2_≥0 controls the smoothness and the scale of the basis vectors; *λ*_1_≥0 controls the sparsity of the coefficient vectors; *λ*_2_≥0 controls the smoothness of the coefficient vectors; and, parameters *t*_1_ and *t*_2_ are boolean variables (0: false, 1: true) which indicate if non-negativity needs to be enforced on ***A*** or ***Y***, respectively. We can call this model *versatile sparse matrix factorization* (VSMF). It can be easily seen that the standard NMF, semi-NMF, and the sparse-NMFs are special cases of VSMF.

We devise the following multiplicative update rules for the VSMF model in the case of *t*_1_=*t*_2_=1 (implemented in function sparsenmf2rule):

(12) $$\begin{array}{ll} \left\{\begin{array}{l} A=A*\frac{XY^{T}}{AYY^{T}+\alpha_{2}A+\alpha_{1}}\;\\ Y=Y*\frac{A^{T}X}{A^{T}AY+\lambda_{2}Y+\lambda_{1}}\; \end{array}\right), & \; \end{array}$$

where, ***A***∗***B*** and $\frac{A}{B}$ are the element-wise multiplication and division operators of matrices ***A*** and ***B***, respectively. Alternatively, we also devise an active-set algorithm for VSMF (implemented in function vsmf). When *t*_1_(or*t*_2_)=1, ***A*** (or ***Y***) can be updated by NNQP (this case is also implemented in sparsenmf2nnqp). When *t*_1_(or*t*_2_)=0, ***A*** (or ***Y***) can be updated using 1_1_QP.

### Kernel-NMF

Two features of a kernel approach are that i) it can represent complex patterns, and ii) the optimization of the model is dimension-free. We now show that NMF can also be kernelized.

The basis matrix is dependent on the dimension of the data, and it is difficult to represent it in a very high (even infinite) dimensional space. We notice that in the NNLS optimization, updating ***Y*** in Equation (10) needs only the inner products ***A***^T^***A***, ***A***^T^***X***, and ***X***^T^***X***. From Equation (4), we obtain ***A***^T^***A***=(***Y***^*‡*^)^T^***X***^T^***XY***^*‡*^, ***A***^T^***X***=(***Y***^*‡*^)^T^***X***^T^***X***. Therefore, we can see that only the inner product ***X***^T^***X*** is needed in the optimization of NMF. Hence, we can obtain the kernel version, *kernel-NMF*, by replacing the inner product ***X***^T^***X*** with a kernel matrix *K*(***X***,***X***). Interested readers can refer to our recent paper [6] for further details. Based on the above derivations, we implemented the kernel semi-NMF using multiplicative update rule (in kernelseminmfrule) and NNLS (in kernelseminmfnnls). The sparse kernel semi-NMFs are implemented in functions kernelsparseseminmfnnls and kernelSparseNMFNNQP which are equivalent to each other. The kernel method of decomposing a kernel matrix proposed in [19] is implemented in kernelnmfdecom.

### Other variants

Ref. [16] proposed the *Convex-NMF*, in which the columns of ***A*** are constrained to be the convex combinations of data points in ***X***. It is formulated as ***X***_±_=***X***_±_***W***_+_***Y***_+_+***E***, where ***M***_±_ indicates that matrix ***M*** is of mixed signs. ***XW***=***A*** and each column of ***W*** contains the convex coefficients of all the data points to get the corresponding column of ***A***. It has been demonstrated that the columns of ***A*** obtained with the convex-NMF are close to the real centroids of clusters. Convex-NMF can be kernelized as well [16]. We implemented the convex-NMF and its kernel version in convexnmfrule and kernelconvexnmf, respectively.

The basis vectors obtained with the above NMFs are non-orthogonal. Alternatively, *orthogonal NMF* (ortho-NMF) imposes the orthogonality constraint in order to enhance sparsity [20]. Its formulation is

(13) $$\begin{array}{lll} & X=ASY+E\;\\ & \text{subject to}\;\;A^{T}A=I,\;YY^{T}=I,\;A,S,Y\geq 0,\; & \; \end{array}$$

where, the input ***X*** is non-negative, ***S*** absorbs the magnitude due to the normalization of ***A*** and ***Y***. Function orthnmfrule is its implementation in our toolbox. Ortho-NMF is very similar with the *non-negative sparse PCA* (NSPCA) proposed in [21]. The disjoint property on ortho-NMF may be too restrictive for many applications, therefore this property is relaxed in NSPCA. Ortho-NMF does not guarantee the maximum-variance property which is also relaxed in NSPCA. However NSPCA only enforces non-negativity on the basis vectors, even when the training data have negative values. We plan to devise a model in which the disjoint property, the maximum-variance property, the non-negativity and sparsity constraints can be controlled on both basis vectors and coefficient vectors.

There are two efficient ways of applying NMF on data containing missing values. First, the missing values can be estimated prior to running NMF. Alternatively, *weighted-NMF*[22] can be directly applied to decompose the data. Weighted-NMF puts a zero weight on the missing elements and hence only the non-missing data contribute to the final result. An expectation-maximization (EM) based missing value estimation during the execution of NMF may not be efficient. The weighted-NMF is given in our toolbox in function wnmfrule.

## Results and discussion

Based on the various implemented NMFs, a number of data mining tasks can be performed via our toolbox. Table 2 lists the data mining functionalities we provide in this level. These mining tasks are also described along with appropriate examples.

**Table 2:** NMF-based data mining approaches

**Function**	**Description**
NMFCluster	Take the coefficient matrix produced by a NMF algorithm, and output the clustering result.
chooseBestk	Search the best number of clusters based on dispersion Coefficients.
biCluster	The biclustering method using one of the NMF algorithms.
featureExtractionTrain	General interface. Using training data, generate the bases of the NMF feature space.
featureExtractionTest	General interface. Map the test/unknown data into the feature space.
featureFilterNMF	On training data, select features by various NMFs.
featSel	Feature selection methods.
nnlsClassifier	The NNLS classifier.
perform	Evaluate the classifier performance.
changeClassLabels01	Change the class labels to be in {0,1,2,⋯,*C*−1} for *C*-class problem.
gridSearchUniverse	A framework to do line or grid search.
classificationTrain	Train a classifier, many classifiers are included.
classificationPredict	Predict the class labels of unknown samples via the model learned by classificationTrain.
multiClassifiers	Run multiple classifiers on the same training data.
cvExperiment	Conduct experiment of k-fold cross-validation on a data set.
significantAcc	Check if the given data size can obtain significant accuracy.
learnCurve	Fit the learning curve.
FriedmanTest	Friedman test with post-hoc Nemenyi test to compare multiple classifiers on multiple data sets.
plotNemenyiTest	Plot the CD diagram of Nemenyi test.
NMFHeatMap	Draw and save the heat maps of NMF clustering.
NMFBicHeatMap	Draw and save the heat maps of NMF biclustering.
plotBarError	Plot Bars with STD.
writeGeneList	Write the gene list into a.txt file.
normmean0std1	Normalization to have mean 0 and STD 1.
sparsity	Calculate the sparsity of a matrix.
MAT2DAT	Write a data set from MATLAB into.dat format in order to be readable by other languages.

### Clustering and bi-clustering

NMF has been applied for clustering. Given data ***X*** with multivariate data points in the columns, the idea is that, after applying NMF on ***X***, a multivariate data point, say ***x***_*i*_ is a non-negative linear combination of the columns of ***A***; that is ***x***_*i*_≈***Ay***_*i*_=*y*_1*i*_***a***_1_+⋯+*y*_*ki*_***a***_*k*_. The largest coefficient in the *i*-th column of ***Y*** indicates the cluster this data point belongs to. The reason is that if the data points are mainly composed with the same basis vectors, they should therefore be in the same group. A basis vector is usually viewed as a cluster centroid or prototype. This approach has been used in [2] for clustering microarray data and in order to discover tumor subtypes. We implemented function NMFCluster through which various NMF algorithms can be selected. An example is provided in exampleCluster file in the folder of our toolbox.

**Figure 1:**
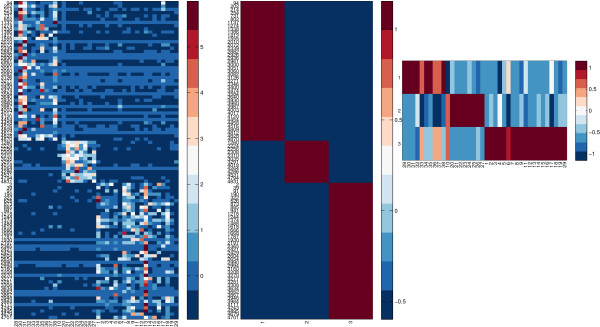
**Heat map of NMF biclustering result.** Left: the gene expression data where each column corresponds to a sample. Center: the basis matrix. Right: the coefficient matrix.

The task of interpreting both the basis matrix and the coefficient is equivalent to simultaneously clustering the rows and columns of matrix ***X***. This is bi-clustering and the interested readers can refer to [23] for an excellent survey on bi-clustering algorithms and to [4] for a bi-clustering method based on NMF. We implemented a bi-clustering approach based on NMF in biCluster function. The bi-clusters can be visualized via the function NMFBicHeatMap. We applied NMF to simultaneously grouping the genes and samples of a leukemia data set [2] which includes tumor samples of three subtypes. The goal is to find strongly correlated genes over a subset of samples. A subset of such genes and a subset of such samples form a bi-cluster. The heat-map is shown in Figure 1. Readers can find the script in exampleBiCluster file of our toolbox.

### Basis vector analysis for biological process discovery

We can obtain interesting and detailed interpretations via an appropriate analysis of the basis vectors. When applying NMF on a microarray data, the basis vectors are interpreted as potential biological processes [3,13,24]. In the following, we give one example for finding biological factors on gene-sample data, and two examples on time-series data. Please note they only serve as simple examples. Fine tuning of the parameters of NMF is necessary for accurate results.

#### First example

We ran our VSMF on the ALLAML gene-sample data of [2] with the settings *k*=3, *α*_1_=0.01, *α*_2_=0.01, *λ*_1_=0, *λ*_2_=0.01, *t*_1_=1, and *t*_2_=1. Next, we obtain 81, 37, and 448 genes for the three factors, respectively. As in [3], we then performed gene set enrichment analysis (GSEA) by applying Onto-Express [25] on each of these sets of genes. Part of the result is shown in Table 3. We can see that the factor-specific genes selected by NMF correspond to some biological processes significantly. Please see file exampleBioProcessGS in the toolbox for details. GSEA can also be done using other tools, such as MIPS [26], GOTermFinder [27], and DAVID [28,29].

**Table 3:** Gene set enrichment analysis using Onto-Express for the factor specific genes identified by NMF

**Factor 1**		**Factor 2**		**Factor 3**	
**biological process**	**p-value**	**biological process**	**p-value**	**biological process**	**p-value**
reproduction (5)	0	response to stimulus (15)	0.035	regulation of bio. proc. (226)	0.009
metabolic process (41)	0	biological regulation (14)	0.048	multi-organism proc. (39)	0.005
cellular process (58)	0			biological regulation (237)	0.026
death (5)	0				
developmental process (19)	0				
regulation of biological process (19)	0				

#### Second example

**Figure 2:**
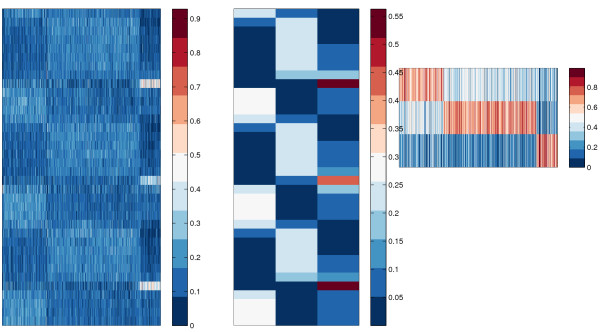
**Heat map of NMF clustering result on yeast metabolic cycle time-series data.** Left: the gene expression data where each column corresponds to a sample. Center: the basis matrix. Right: the coefficient matrix.

**Figure 3:**
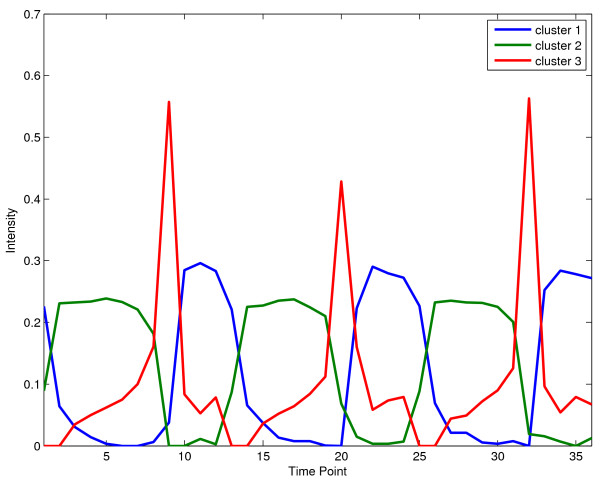
Biological processes discovered by NMF on yeast metabolic cycle time-series data.

We used NMF to cluster a time-series data of yeast metabolic cycle in [30]. Figure 2 shows the heat-map of NMF clustering, and Figure 3 shows the three basis vectors. We used nmfnnls function to decompose the data and NMFHeatMap to plot the heat-map. The detailed script is given in the exampleBioProcessTSYeast file in the toolbox. We can clearly see that the three periodical biological processes corresponds exactly to the Ox (oxidative), R/B (reductive, building), and R/C (reductive, charging) processes discovered in [30].

#### Third example

**Figure 4:**
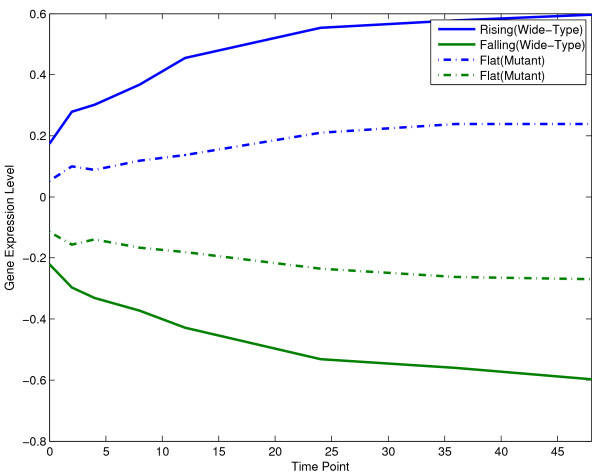
Biological processes discovered by NMF on breast cancer time-series data.

We used NMF to factorize a breast cancer time-series data set, which includes wild type MYCN cell lines and mutant MYCN cell lines [31]. The purpose of this example is to show that NMF is a potential tool to finding cancer drivers. One basic methodology is in the following. First, basis vectors are produced applying NMF on a time-series data. Then factor-specific genes are identified by computational or statistical methods. Finally, the regulators of these factor-specific genes are identified from any prior biological knowledge. This data set has 8 time points (0, 2, 4, 8, 12, 24, 36, 48 hr.). The zero time point is untreated and samples were collected at the subsequent time points after treatment with 4-hydroxytamoxifen (4-OHT). In our computational experiment, we use our VSMF implementation (function vsmf). We set *k*=2. Because this data set has negative values we set *t*_1_=0 and *t*_2_=1. We set *α*_1_=0.01, *α*_2_=0, *λ*_1_=0, and *λ*_2_=0.01. The basis vectors of both wild-type and mutant data are compared in Figure 4. From the wild-type time-series data, we can successfully identify two patterns. The rising pattern corresponds to the induced signature and the falling pattern corresponding to the repressed signature in [31]. It is reported in [31] that the MYC target genes contributes to both patterns. From the mutant time-series, we can obtain two flat processes, which are reasonable. The source code of this example can be found in exampleBioProcessMYC. We also recommend the readers to see the methods based on matrix decompositions which are proposed in [13,32] and devised for identifying signaling pathways.

### Basis vector analysis for gene selection

The columns of ***A*** for a gene expression data set are called *metasamples* in [2]. They can be interpreted as biological processes, because their values imply the activation or inhibition of some the genes. Gene selection aims to find marker genes for disease prediction and to understand the pathways they contribute to. Rather than selecting genes on the original data, the novel idea is to conduct gene selection on the metasamples. The reason is that the discovered biological processes via NMF are biologically meaningful for class discrimination in disease prediction, and the genes expressed differentially across these processes contribute to better classification performance in terms of accuracy. In Figure 1 for example, three biological processes are discovered and only the selected genes are shown. We have implemented the information-entropy-based gene selection approach proposed in [3] in function featureFilterNMF. We give an example on how to call this function in file exampleFeatureSelection. It has been reported that it can select meaningful genes, which has been verified with gene ontology analysis. Feature selection based on supervised NMF will also be implemented.

### Feature extraction

Microarray data and mass spectrometry data have tens of thousands of features but only tens or hundreds of samples. This leads to the issues of *curse of dimensionality*. For example, it is impossible to estimate the parameters of some statistical models since the number of their parameters grow exponentially as the dimension increases. Another issue is that biological data are usually noisy; which crucially affects the performances of classifiers applied on the data. In cancer study, a common hypothesis is that only a few biological factors (such as the oncogenes) play a crucial role in the development of a given cancer. When we generate data from control and sick patients, the high-dimensional data will contain a large number of irrelevant or redundant information. Orthogonal factors obtained with *principal component analysis* (PCA) or *independent component analysis* (ICA) are not appropriate in most cases. Since NMF generates non-orthogonal (and non-negative) factors, therefore it is much reasonable to extract important and interesting features from such data using NMF. As mentioned above, training data ***X***_*m*×*n*_, with *m* features and *n* samples, can be decomposed into *k* metasamples ***A***_*m*×*k*_ and ***Y***_*k*×*n*_, that is

(14) $$X\approx AY_{\text{tr}},\text{subject to}A,Y_{\text{tr}}\geq 0,$$

where, ***Y***_tr_ means that ***Y*** is obtained from the training data. The *k* columns of ***A*** span the *k*-dimensional *feature space* and each column of ***Y***_tr_ is the representation of the corresponding original training sample in the feature space. In order to project the *p* unknown samples ***S***_*m*×*p*_ into this feature space, we have to solve the following non-negative least squares problem:

(15) $$S\approx AY_{\text{uk}},\text{subject to}Y_{\text{uk}}\geq 0,$$

where, ***Y***_uk_ means the ***Y*** is obtained from the unknown samples. After obtaining ***Y***_tr_ and ***Y***_uk_, the learning and prediction steps can be done quickly in the *k*-dimensional feature space instead of the *m*-dimensional original space. A classifier can learn over ***Y***_tr_, and then predicts the class labels of the representations of unknown samples, that is ***Y***_uk_.

From the aspect of interpretation, the advantage of NMF over PCA and ICA is that the metasamples are very useful in the understanding of the underlying biological processes, as mentioned above.

We have implemented a pair of functions featureExtractionTrain and featureExtractionTest including many linear and kernel NMF algorithms. The basis matrix (or, the inner product of basis matrices in the kernel case) is learned from the training data via the function featureExtractionTrain, and the unknown samples can be projected onto the feature space via the function featureExtractionTest. We give examples of how to use these functions in files exampleFeatureExtraction and exampleFeatureExtractionKernel.

**Figure 5:**
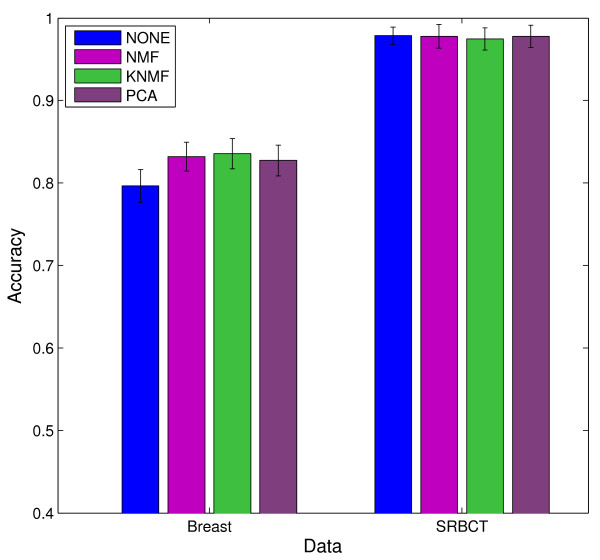
Mean accuracy and standard deviation results of NMF-based feature extraction on SRBCT data.

Figure 5 shows the classification performance of SVM *without* dimension reduction and SVM *with* dimension reduction using linear NMF, kernel NMF with *radial basis function* (RBF) kernel, and PCA on two data sets, SRBCT [33] and Breast [34]. Since ICA is computationally costly, we did not include it in the comparisons. The bars represent the averaged 4-fold cross-validation accuracies using *support vector machine* (SVM) as classifier over 20 runs. We can see that NMF is comparable to PCA on SRBCT, and is slightly better than PCA on Breast data. Also, with only few factors, the performance after dimension reduction using NMF is at least comparable to that without using any dimension reduction. As future work, supervised NMF will be investigated and implemented in order to extract discriminative features.

### Classification

If we make the assumption that every unknown sample is a sparse non-negative linear combination of the training samples, then we can directly derive a classifier from NMF. Indeed, this is a specific case of NMF in which the training samples are the basis vectors. Since the optimization process within NMF is a NNLS problem, we call this classification approach the *NNLS classifier*[35]. A NNLS problem is essentially a quadratic programming problem as formulated in Equation (9), therefore, only inner products are needed for the optimization. We thus can naturally extend the NNLS classifier to kernel version. Two features of this approach are that: i) the sparsity regularization help avoid overfitting the model; and ii) the kernelization allows a dimension-free optimization and also linearizes the non-linear complex patterns within the data. The implementation of the NNLS classifier is in file nnlsClassifier. Our toolbox also provides many other classification approaches including SVM classifier. Please see file exampleClassification for demonstration. In our experiment of 4-fold cross-validation, accuracies of 0.7865 and 0.7804 are respectively obtained with linear and kernel (RBF) NNLS classifier on Breast data set. They achieved accuracies of 0.9762 and 0.9785, respectively, over SRBCT data.

Biological data are usually noisy and sometimes contain missing values. A strength of the NNLS classifier are that it is robust to noise and to missing values, making NNLS classifiers quite suitable for classifying biological data [35].

**Figure 6:**
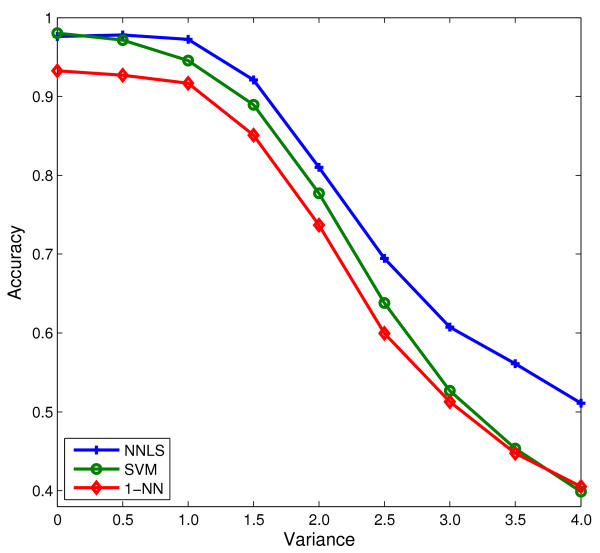
The mean accuracy results of NNLS classifier for different amount of noise on SRBCT data.

In order to show its robustness to noise, we added a Gaussian noise of mean 0 and variance from 0 to 4 with increment 0.5 on SRBCT. Figure 6 illustrates the results of NNLS, SVM, and *1-nearest neighbor* (1-NN) classifiers using this noisy data. It can be seen that as the noise increases, NNLS outperforms SVM and 1-NN significantly.

**Figure 7:**
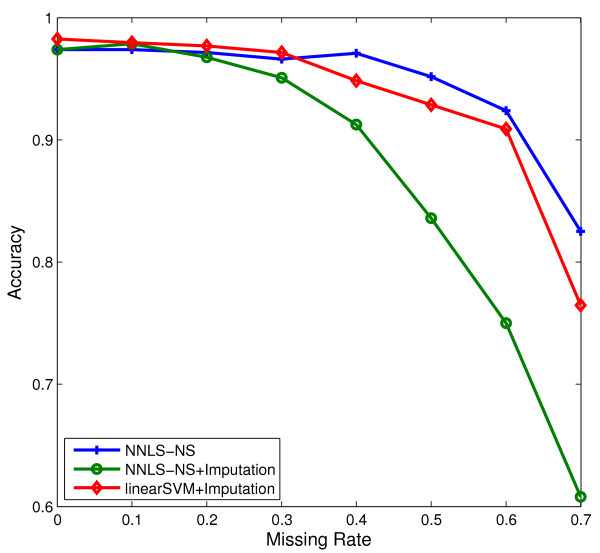
The mean accuracy results of NNLS classifier for different missing value rates on SRBCT data.

To deal with the missing value problem, three strategies are usually used: incomplete sample or feature removal, missing value imputation (i.e., estimation), and ignoring missing values. Removal methods may delete important or useful information for classification and particularly when there is a large percentage of missing values in the data. Imputation methods may create false data depending on the magnitude of the true estimation errors. The third method ignores using the missing values during classification. Our approach in dealing with the missing value problem is also to ignore them. The NNLS optimization needs only the inner products of pairs of samples. Thus, when computing the inner product of two samples, say ***x***_*i*_ and ***x***_*j*_, we normalize them to have unit *l*_2_-norm using only the features present in both samples, and then we take their inner product. As an example, we randomly removed between 10% to 70% of data values in STBCT data. Using such incomplete data, we compared our method with the zero-imputation method (that is, estimating all missing values as 0). In Figure 7, we can see that the NNLS classifier using our missing value approach outperforms the zero-imputation method in the case of large missing rate. Also, the more sophisticated *k*-nearest neighbor imputation (KNNimpute) method [36] will fail on data with in high percentage of missing values.

### Statistical comparison

**Figure 8:**
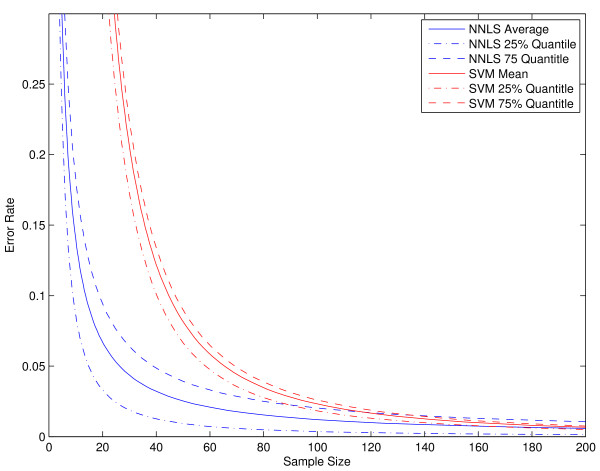
The fitted learning curves of NNLS and SVM classifiers on SRBCT data.

The toolbox provides two methods for statistical comparisons and evaluations of different methods. The first is a two-stage method proposed in [37]. The importance of this method is that it can estimate the data-size requirement for attaining a significant accuracy and extrapolate the performance based on the current available data. Generating biological data is usually very expensive and thus this method can help researchers to evaluate the necessity of producing more data. At the first stage, the minimum data size required for obtaining a significant accuracy is estimated. This is implemented in function significantAcc. The second stage is to fit the learning curve using the error rates of large data sizes. It is implemented in function learnCurve. In our experiments, we found that the NNLS classifier usually requires fewer number of samples for obtaining a significant accuracy. For example on SRBCT data, NNLS requires only 4 training samples while SVM needs 19 training samples. The fitted learning curves of NNLS and SVM classifiers are shown in Figure 8. We provide an example of how to plot this figure in file exampleFitLearnCurve.

**Figure 9:**
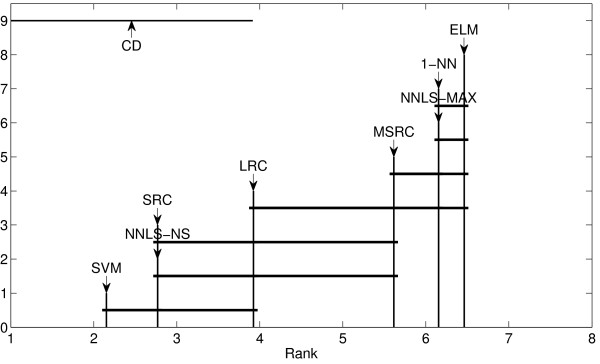
**Nemenyi test comparing 8 classifiers over 13 high dimensional biological data (*α=0.05*).**

The second method is the nonparametric Friedman test coupled with post-hoc Nemenyi test to compare multiple classifiers over multiple data sets [38]. It is difficult to draw an overall conclusion if we compare multiple approaches in a pairwise fashion. Friedman test has been recommended in [38] because it is simple, safe and robust, compared with parametric tests. It is implemented in function FriedmanTest. The result can be presented graphically using the *crucial difference* (CD) diagram as implemented in function plotNemenyiTest. CD is determined by significant level *α*. Figure 9 is an example of the result of the Nemenyi test for comparing 8 classifiers over 13 high dimensional biological data sets. This example can be found in file exampleFriedmanTest. If the distance of two methods is greater than the CD then we conclude that they differ significantly.

## Conclusions

In order to address the issues of the existing NMF implementations, we propose a NMF Toolbox written in MATLAB, which includes a basic NMF optimization level and an advanced data mining level. It enable users to analyze biological data via NMF-based data mining approaches, such as clustering, bi-clustering, feature extraction, feature selection, and classification.

The following are the future works planned in order to improve and augment the toolbox. First, we will include more NMF algorithms such as nsNMF, LS-NMF, and supervised NMF. Second, we are very interested in implementing and speeding up the Bayesian decomposition method which is actually a probabilistic NMF introduced independently in the same period as the standard NMF. Third, we will include more statistical comparison and evaluation methods. Furthermore, we will investigate the performance of NMF for denoising and for data compression.

## Availability and requirements

**Project name:** The NMF Toolbox in MATLAB**Project home page:**https://sites.google.com/site/nmftool and http://cs.uwindsor.ca/~li11112c/nmf**Operating system(s):** Platform independent**Programming language:** MATLAB**Other requirements:** MATLAB 7.11 or higher**License:** GNU GPL Version 3**Any restrictions to use by non-academics:** Licence needed

## Competing interests

The authors declare that they have no competing interests.

## Authors’ contributions

YL did comprehensive survey on NMF, implemented the toolbox, and drafted this manuscript. AN supervised the whole project, provided constructive suggestions on this toolbox, and wrote the final manuscript. All authors read and approved the final manuscript.
